# Objective Comparison between Platelet Rich Plasma Alone and in Combination with Physical Therapy in Dogs with Osteoarthritis Caused by Hip Dysplasia

**DOI:** 10.3390/ani10020175

**Published:** 2020-01-21

**Authors:** Belén Cuervo, Mónica Rubio, Deborah Chicharro, Elena Damiá, Angelo Santana, José María Carrillo, Ayla Del Romero, José Manuel Vilar, José Joaquín Cerón, Joaquín Jesús Sopena

**Affiliations:** 1 Bioregenerative Medicine and Applied Surgery Research Group, Departamento de medicina y cirugía animal, Facultad de veterinaria, Universidad Cardenal Herrera-CEU, CEU Universities, 46115 Valencia, Spain; belen.cuervo@uchceu.es (B.C.); mrubio@uchceu.es (M.R.); debora.chicharro@uchceu.es (D.C.); elena.damia@uchceu.es (E.D.); jcarrill@uchceu.es (J.M.C.); ayla.delromero@uchceu.es (A.D.R.); jsopena@uchceu.es (J.J.S.); 2 García Cugat Foundation CEU-UCH Chair of Medicine and Regenerative Surgery, 08006 Barcelona, Spain; 3 Department of Mathematics, Universidad de Las Palmas de Gran Canaria, Campus de Tafira, 35018 Las Palmas, Spain; angelo.santana@ulpgc.es; 4 Department of Animal Pathology, Instituto Universitario de Investigaciones Biomédicas y Universitarias, Universidad de Las Palmas de Gran Canaria, 35416 Trasmontaña S/N. Arucas, Spain; 5 Interdisciplinary Laboratory of Clinical Analysis (Interlab-UMU), Veterinary School, Campus of Excellence Mare Nostrum, University of Murcia, Espinardo, 30100 Murcia, Spain

**Keywords:** plasma rich in growth factors, platelet rich plasma, osteoarthritis, canine, physical therapy, biomarkers

## Abstract

**Simple Summary:**

Finding successful treatments against osteoarthritis without secondary effects and objectively assessing their effectiveness in dogs is always challenging. In this sense, the aim of this study was to objectively assess the efficacy of platelet rich plasma alone and in combination with physical therapy by using a force platform in dogs with hip osteoarthritis. Dogs receiving only the platelet rich plasma treatment showed a significant improvement in limb function, although the effect decreased after 180 days; in contrast, the combined therapy maintained the maximum level of efficacy throughout the study period. Our study proved that physical therapy does not increase the level of efficacy of platelet rich plasma therapy but objectively contributes to prolonging its effect for more than this time period.

**Abstract:**

Osteoarthritis (OA) is one of the most significant joint diseases worldwide. There are different therapies for OA treatment, and a relatively new strategy is the use of plasma rich in growth factors (PRGF), a platelet rich plasma (PRP) derivative. The objective of this study was to objectively assess the efficacy and duration of the effect of an intraarticular injection of PRGF and a combination of PRGF + physical therapy. The objective assessment was provided using a force platform. The obtained parameters were peak vertical force (PVF) and vertical impulse (VI). A total of 24 dogs with lameness and pain associated to OA attributable to bilateral hip dysplasia were included in the study. Animals were divided into two study groups and evaluated at baseline and at 30, 90, and 180 days after intraarticular PRGF or PRGF + physical therapy. Significant differences were observed at every checkpoint with respect to basal time in both groups. However, after 180 days, the PRGF group showed a decrease in PVF and VI with respect to the values obtained at 90 days. However, the PRGF + physical therapy group maintained increased values of both PVF and VI values during the 180-day study period.

## 1. Introduction

Osteoarthritis (OA) is a common degenerative joint disease affecting articular cartilage in both human and veterinary medicine due to an increased life expectancy in people and animals [1,2,3]. It is characterized by progressive deterioration and loss of articular cartilage with subchondral bone affection, the formation of osteophytes, the thickening of the joint capsule, and synovitis. These lesions lead to discomfort and pain in the affected joint, causing functional limitation; this limitation could be quantified, given that kinetic parameters such as peak vertical force (PVF) and vertical impulse (VI) show decreased values. Moreover, this is a disabling disease that decreases patients’ quality of life and commonly affected joints are the elbow, stifle, and hip joints [3,4].

Hip dysplasia (HD) is the most common developmental orthopedic condition in dogs and is highly breed dependent [5]. HD can be defined as the abnormal development of the hip joint, resulting in coxofemoral laxity [6]. Disease progression results in degenerative and inflammatory changes characteristic of osteoarthritis (OA) [7]. A conservative strategy against HD includes physical therapy (PT). The approach should be multimodal and focused on improving function, reducing clinical signs of pain, improving hip movement and strength, and potentially slowing or minimizing the progression of OA [8].

There is a variety of medical therapies used to treat OA. Until recently, the aim of these therapies was to decrease the pain and discomfort of patients by using analgesics and nonsteroidal anti-inflammatory drugs (NSAIDs) [9]. Currently, the most innovative therapies aim to prevent or delay joint degeneration, stimulating the repair process of the damaged area. As a result, a larger number of studies are being conducted to prove the effectiveness of regenerative therapies, such as using platelet rich plasma (PRP) [10].

PRP is described as an autologous blood-derived biological product obtained after centrifugation. It contains a high number of platelets in a small volume of plasma, with an approximate pH of 6.6, and due to its autologous origin, it is considered a safe product [11]. Platelets in PRP can be activated by adding calcium chloride to stimulate the release of the granule’s contents. PRP contains significant amounts of cytokines and growth factors (GF), which are a group of soluble and diffusible polypeptide substances that regulate growth, proliferation, differentiation, and metabolism of cells [12,13,14]. Some of the GF involved in chondrogenesis and cartilage regeneration are platelet-derived growth factors (PDGF), transforming growth factors β (TGF-β), insulin-like growth factors (IGF-1), and fibroblast growth factors (FGF) [15,16]. There are numerous PRP variants depending on their different characteristics; the plasma rich in growth factors (PRGF), for example, has a moderate platelet concentration and an absence of leukocytes [17].

In addition to GF, PRP also contains several proteins that play an important role during tissue repair and regeneration, such as fibrinogen, fibronectin, and vitronectin. They allow cell and other molecule adhesion, which is useful for cell conduction, acting as a support "matrix" for tissue repair [18,19]. Assessing the duration of effectiveness of PRP has been controversial, mainly because study designs use different assessing methodologies and different protocols of PRP obtention [20]. Studies in human species show that pain relief lasts for more than six months [21]. On the contrary, one study in dogs showed how the PRP effect disappeared after this same study period [22]. This fact highlights the need to develop objective methods to assess the PRP level of efficacy and duration of effect [23].

Several different approaches are currently available for diagnosing and evaluating the responses to treatments. In this sense, biomechanics and specifically kinetic analysis using a force platform has been considered to be the “gold standard” for limb function assessment. This methodology has successfully assessed different treatments against OA [24,25].

As a result, the initial hypothesis of the present study was that, even though a single intraarticular infiltration of a PRP infusion would improve the limitations in limb functionality associated with OA, the combination of a PRP infusion with a physical rehabilitation program would significantly improve the effect in terms of level of efficacy and duration. Therefore, the objective of our study was to evaluate the effectiveness of intraarticular PRP infiltration and PRP + physical rehabilitation through objective kinetic parameters, such as PVF and VI.

## 2. Materials and Methods

### 2.1. Animals

The current clinical study was carried out with 24 client-owned dogs of different breeds and ages, both males and females, presenting with OA caused by bilateral HD. All animal owners included in the study signed a written consent after having been notified of the relevant project information. The protocol was approved by the ethics committee of the University of Las Palmas de Gran Canaria. Descriptive data for each patient is detailed in Table 1a,b.

Main descriptive results for the dogs used in this study are summarized in Table 2.

Dogs included in the present study weighed more than 30 kg. All the animals showed a 3–4 grade of lameness in a scale of four grades (obvious lameness at walk and trot). To confirm the presence of moderate to severe OA in each animal, a complete orthopedic examination was performed with X-rays under sedation. Radiographic signs of OA were evaluated, such as the presence of subchondral bone sclerosis, bone remodeling, and the presence of osteophytes or diminished joint space [26,27]. Except for OA, the animals could not present any other pathology, therefore requiring each dog to undergo a complete clinical examination and complete hematology, as well as a serum biochemistry and an endocrine and serology panel.

Two groups of 12 dogs were formed: One study group (PRGF) receiving a single intraarticular dosage of PRGF bilaterally and a second group (PRGF + PT) receiving a combined treatment of PRGF and a PT program.

### 2.2. PRGF Preparation-Inoculation

PRGF^®^-Endoret^®^ technology (BTI Biotechnology Institute, Alava, Spain) was used to obtain autologous preparation of PRP. The procedure has been previously published [17], but briefly: 

The blood was collected from the external jugular vein of each dog under sterile conditions in Vacutainer sodium citrate 3.8% tubes (Blood-Collecting Tubes^®^, BTI Biotechnology Institute, Alava, Spain). Next, the tubes were centrifuged at 460× *g* for 8 min (PRGF^®^ System III, Biotechnology Institute, Alava, Spain) to separate the different blood phases. The fraction located immediately above the buffy coat (white fraction) corresponds to PRGF. The extraction procedure of the plasma fractions was carried out under the maximum conditions of sterility in the laminar flow cabinet and always by the same individual. Once the different plasma fractions were extracted, a total of 2 mL of PRGF was infiltrated into both affected joints by conventional arthrocentesis. Prior to infiltration, PRGF was activated by adding 5% of calcium chloride (CaCl_2_ 10%) to activate platelets for growth factor release.

### 2.3. PT Program

The aim of the rehabilitation program was to increase the joint range of motion, as well as potentiate the muscular strength of the hind limbs and the physical endurance of the dogs. This part of the treatment had an individual approach; in this sense, the rehabilitation program intensity was modified gradually as each animal progressed until all of the tasks below were accomplished. The activities should be performed once daily and should be maintained until the end of the study; these were:

(1) Warm-up, consisted in 5’ at slow walk in a straight, horizontal walkway.
(2) Sit-to-stand, 15 times.
(3) Dancing exercises, 5’ raising the forelimbs off the ground and walking the dog forward and backward.
(4) Incline walking, 10’ at regular walk.
(5) Cool down, again 5’ at slow walk in a straight, horizontal walkway.

### 2.4. Force Platform Measurement

A single platform was placed in the center of a 7 m runway covered by a rubber mat. Dogs were leash guided at a walk over the force platform by the same handler. Walk velocity was measured by a motion sensor (PS-2103a, Pasco, CA, USA) in order to ensure that animals walked homogeneously within a narrow variation of velocity (1.6 ± 0.5 m/s) and acceleration (≤0.5 m/s^2^). The sampling frequency was set to 250 Hz. A total of five valid trials were obtained from each dog. A trial was considered valid when the limb fully contacted the force platform, with the dog walking next to the handler without pulling on the leash. 

The software DataStudio (Pasco, CA, USA) was used for the acquisition, numerical conversion, and storage of data. Both affected limbs were recorded at day (D) 0, 30, 90, and 180 post-treatment. Finally, the obtained PVF (Newtons) and VI (Newtons x second) values were normalized relative to body weight (%).

Although each dog had bilateral dysplasia, only the measurements obtained from the more lame limbs (lesser PVF) were considered reliable in order to avoid a possible bias caused by inconsistent weight redistribution to the less affected contra-lateral hind limb. For this reason, only data from the more lame limbs were statistically compared.

### 2.5. Statistical Analysis

In our study design, the dog was considered a blocking factor, while time from the start of treatment (D0, D30, D90, D180) and treatment (PRGF, PRGF + PT) were considered experimental factors, given that the aim of the study was to determine if there were systematic differences in the % of PVF or % of VI between both treatment strategies among four fixed moments in time: D0, D30, D90, and D180.

For the data analysis, a linear mixed-effects model was considered: The experimental factors (time and treatment) were fixed effects factors, while the blocking factor (dog) was a random-effects factor. The model was as follows:

$$y_{ijkl}=\mu +\alpha_{i}+\beta_{j}+{\left(\alpha \beta \right)}_{ij}+b_{k}+b_{jk}+\varepsilon_{ijkl}$$

where:

- $y_{ijkl}$ is the l-th measure (l=1,2,…,5) of PVF/VI of the dog k (k=1…12) in the month j=0 (D0), 1 (D30), 3 (D90), and 6 (D180), being the dog assigned to treatment i=PRGF/PRGF+PT.
- μ is the grand mean of the % PVF/VI for all data.
- $\alpha_{i}$ is the (fixed) effect of the treatment i.
- $\beta_{j}$ is the fixed effect of time j.
- ${\left(\alpha \beta \right)}_{ij}$ represents the interaction between the treatment i and time j.
- $b_{k}$ is the (random) effect of the dog k. Values of $b_{k}$ are supposed to be normally distributed with a mean 0 and standard deviation $\sigma_{D}$. So, $\sigma_{D}$ is the variability in the response due to the dog.
- $b_{jk}$ is the (random) effect of the k-th dog in the j-th time of observation. Values of $b_{jk}$ are supposed to be normally distributed with a mean 0 and standard deviation $\sigma_{TD}$. This term allows for the possibility of nonparallel responses in all dogs of the same group as time progresses (i.e., the possibility of dogs improving in the same time period as others worsen in the same group).
- $\varepsilon_{ijkl}$ is the residual in the measure ijkl. This variable is assumed to be also normally distributed with a mean 0 and standard deviation σ.

Parameters in this model were estimated using the package nlme in the R statistical software. A one-way ANOVA test was used to verify the existence of significant differences between treatments or between times. When significant differences were detected, the post-hoc Tukey multiple comparison test was used to compare fixed effects. For assessing the validity of the model, a Shapiro–Wilk test was applied for testing normality of the residuals, and a Levene test was applied for testing homoscedasticity.

## 3. Results

No significant differences between groups were found in weight (*p* = 0.56) or in age (*p* = 0.10). Mean values (± SD) of PVF and VI in PRGF and PRGF + TP groups at D0, D30, D90, and D180 are summarized in Table 3.

**Figure 1:**
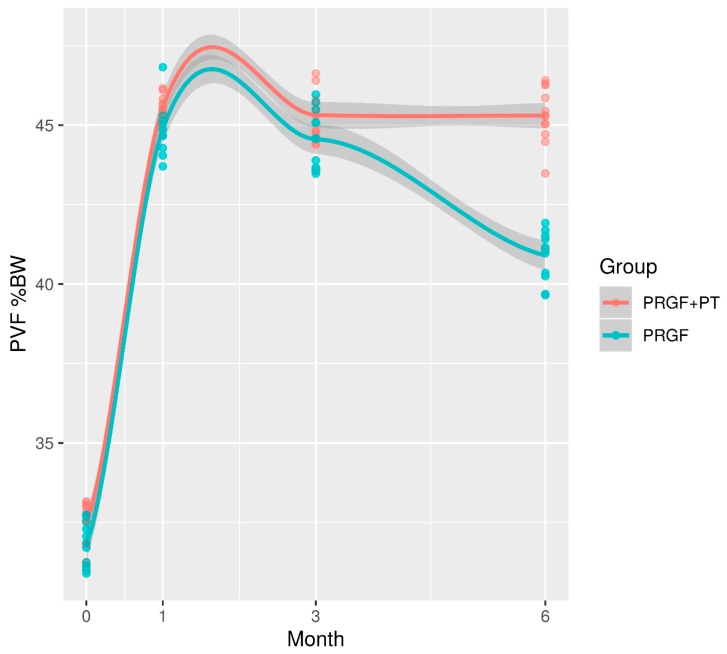
Evolution of PVF (normalized to body weight) in both groups during the 180 days of the study. PVF: Peak vertical force; PRGF: Plasma rich in growth factors; PT: Physical therapy.

**Figure 2:**
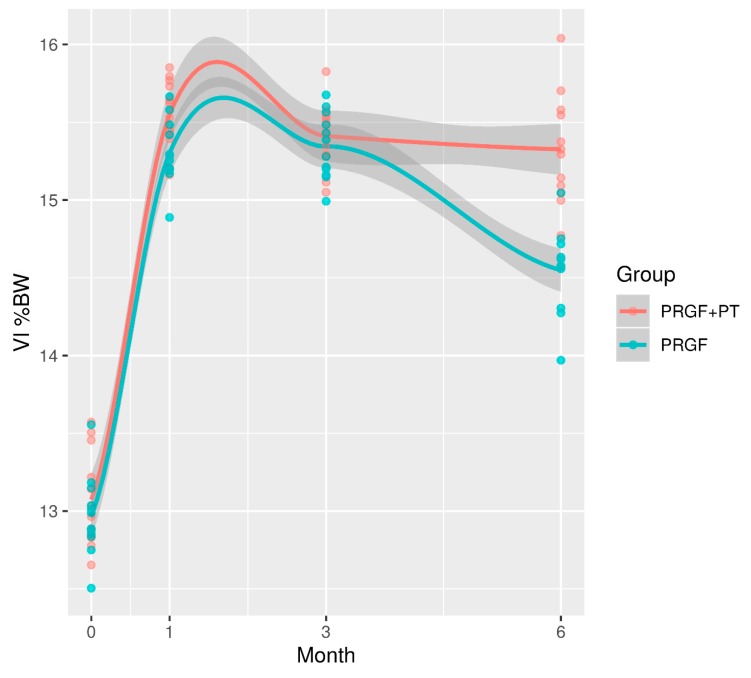
Evolution of VI (normalized to body weight) in both groups during the 180 days of the study. PRGF: Plasma rich in growth factors; PT: Physical therapy; VI: Vertical impulse.

In both groups, PVF and VI significantly increased (*p* ≤ 0.001) at D30 when compared with D0. This difference remained significant in all the remaining time periods. When both groups were compared, no significant differences were found (*p* ≥ 0.005) at D0, D30, and D90; however, PVF and VI at D180 in the PRGF group was 4.4% and 0.78% lower (*p* ≤ 0.001), respectively, when compared with the PRGF + PT group (Figure 1 and Figure 2).

In Figure 1 and Figure 2, each point corresponds with the mean value (PVF or VI) for each dog. A smoothing curve obtained by local polynomial regression has been superimposed on each treatment point to better represent the average trend over time. The shaded region is the 95% confidence band for this average trend. PVF and VI in the PGRF group decreased between D90 and D180. In both groups, PVF and VI residuals were respectively normal (*p* = 0.24, *p* = 0.16) and homoscedastic (*p* = 077, *p* = 0.75).

## 4. Discussion

In agreement with our hypothesis, PGRF therapy, a PRP derivative, is effective in improving limb function in dogs with OA, although this effect decreased after 180 days; however, when combined with PT, the effect is extended in terms of time, maintaining its efficacy beyond this period. To the authors knowledge, this is the first study collecting objective data regarding the effect of physical therapy in dogs with hip OA.

Currently, adequate treatment in OA remains a daunting clinical challenge, despite the advances in medicine, since cartilage has a limited regenerative capacity. Usually, the treatments for this pathology only reach temporary clinical or functional improvements; for this reason, the main objectives when treating this pathology are controlling pain, improving functionality, and stopping the progression of the disease [28].

Even though most of the published research and clinical studies show positive results using PRP for OA treatment, this therapy remains unpredictable due to the significant heterogeneity between studies and the variability in PRP preparations. When compared with a “generic” PRP, where the effect in stifle OA disappeared after 180 days [21], PRGF maintained its effect for more than the six-month period, although the effect decreased. In order to obtain consistent results and conclusions, we decided to use PRGF because it is a validated and registered product with demonstrated effectiveness and minimal adverse effects [28,29].

There is no current consensus in regards to a standard regimen. In our study, as well as in previous studies [25,30], we observed at a single intraarticular application of PRGF functional limb deficit improvement presented by canine patients derived from osteoarthritic pathology until D180 after treatment when compared with D0. 

Muscle atrophy is often seen in animals with hip dysplasia; this occurs because the muscles most vulnerable to disuse atrophy are the postural muscles, extensor muscles, and muscles that cross a single joint, such as the hip. Unfortunately, even if the muscle mass is recovered, peak force could still be reduced by 50% [31]. In addition, muscular weakness predisposes OA progression and clinical signs in humans [32] and compromises joint stability [33]. In light of this research, we reasoned that the PT protocol should not only consist of mobilization exercises, but also exercises that promote muscle strengthening. In this way, muscles not only increase their volume, as demonstrated by other studies [34], but also increase their power [35]. Since muscle regeneration is highly age-dependent, with a high potential for success when dogs are young [36], it is necessary for remobilization and strengthening exercises in adult/senior dogs to start as soon as they are diagnosed.

The usefulness of kinetic, objective, and quantifiable assessments of the effectiveness of different therapeutical strategies against OA have been previously proven [37,38,39]. In our study, PVF and VI objectively show how both PRGF and PRGF + PT therapies equally improve limb function, although the duration of the maximal effect was significantly different. Some studies also found improvement in limb function in dogs when PT is combined with tibial plateau leveling osteotomy (TPLO) surgery due to cranial cruciate rupture (CCLR) [34,35,40]. Although some of the previous studies also used objective data from force platform analysis, conclusions cannot be extrapolated given the difference in the affected joint (stifle), and the primary treatment was surgical, not conservative. Nonetheless, few clinicians are prone to include rehabilitation techniques in the treatment process because of its cost or their lack of knowledge in the field, among others [41]. 

Our study has some limitations. The first limitation is that it only evaluated the effect of a single PRGF injection. It would have been interesting to compare the effect of one injection with more than one injection to assess if duration and effectiveness differed. However, in our opinion, the objective to prove PT as a beneficial co-adjuvant to other therapies, which is the case of a single dosage of PRGF, was reached. The second and last limitation is the duration of the evaluation. After analyzing the results, six months appears to be long enough to evaluate the treatment’s effectiveness, in keeping with previous studies [42]. By extending the duration of the study, and if PT is maintained, it would determine when the PRGF effect disappears; however, that was outside the scope of our objectives. In addition, consideration should be made that the animals included in this study belong to owners; it is probable that increasing the length of the study would negatively affect the owners commitment and, therefore, the number of animals that could be properly evaluated.

## 5. Conclusions

A single intraarticular infiltration of PRGF in canine patients with bilateral hip dysplasia due to OA is effective in relieving pain and improving limb function. The combination of a PT program along with a PRGF treatment allows to maintain the maximum effect for more than six months, which is not the case if dogs are treated with PRGF alone.

## Figures and Tables

**Table 1:** (**a**). Descriptive data of each patient included in the group PRGF. (**b**) Descriptive data of each patient included in the group PRGF + PT.

**(a)**				
**Dog**	**Breed**	**Gender**	**Weight (kg)**	**Age (Months)**
1	German shepherd	M	37.00	60
2	Crossbreed	F	32.50	96
3	Siberian husky	M	30.10	94
4	Labrador retriever	M	31.50	120
5	Crossbreed	M	43.80	72
6	Rottweiler	M	55.00	120
7	Crossbreed	M	36.50	108
8	Crossbreed	F	44.20	106
9	Labrador retriever	M	31.40	56
10	Caucasian shepherd	F	61.00	105
11	Golden retriever	F	33.00	120
12	Labrador retriever	M	32.30	108
**(b)**				
1	Labrador retriever	M	35.80	132
2	Crossbreed	M	31	60
3	Belgian Shepherd	F	32.2	71
4	German shepherd	M	36.5	87
5	Crossbreed	M	30	43
6	Canarian presa	M	59	99
7	Crossbreed	F	31.7	123
8	Rottweiler	F	38.4	59
9	Rhodesian ridgeback	F	36.7	74
10	Canarian presa	M	51	52
11	Golden retriever	M	37	87
12	Labrador retriever	F	36	47

PRGF: Plasma rich in growth factors; PT: Physical therapy; M: Male; F: Female.

**Table 2:** Descriptive results for the dogs included in this study.

	PRGF	PRGF + PT
Weight	39.02 ± 10.07	37.94 ± 8.58
Age (months)	97.08 ± 22.68	77.83 ± 28.85
Males	8	7
Females	4	5

PRGF: Plasma rich in growth factors; PT: Physical therapy.

**Table 3:** Mean ± SD values for PVF and VI for PRGF and PRGF + PT groups at the different checking periods.

	Group	D0	D30	*Dif*	D90	*Dif*	D180	*Dif*
PVF	PRGF	31.77 ± 1.16	44.89 ± 1.26	13.13 †	44.56 ± 1.25	12.79% †	40.90 ± 1.26	9.13 † *
	PRGF + PT	32.42 ± 1.08	45.56 ± 0.84	13.14 †	45.32 ± 1.10	12.9% †	45.30 ± 1.16	12.88 † *
VI	PRGF	12.97 ± 0.37	15.30 ± 0.35	2.33 †	15.34 ± 0.39	2.37% †	14.55 ± 0.41	1.58 † *
	PRGF + PT	13.07 ± 0.45	15.55 ± 0.35	2.47 †	15.41 ± 0.32	2.33% †	15.33 ± 0.51	2.25 † *

*Dif* refers to percent of difference with D0; † refers to a significant difference with D0; * refers to significant differences between both groups at the same time period. PVF: Peak vertical force; VI: Vertical impulse; PRGF: Plasma rich in growth factors; PT: Physical therapy.

## References

[1] Hunter D.J. (2013). Osteoarthritis. Rheum. Dis. Clin. N. Am..

[2] Rychel J.K. (2010). Diagnosis and treatment of osteoarthritis. Top Companion Anim. Med..

[3] Malek S., Sample S.J., Schwartz Z., Nemke B., Jacobson P.B., Cozzi E.M., Schaefer S.L., Bleedorn J.A., Holzman G., Muir P. (2012). Effect of analgesic therapy on clinical outcome measures in a randomized controlled trial using client-owned dogs with hip osteoarthritis. BMC Vet. Res..

[4] Juni P., Reichenbach S., Dieppe P. (2006). Osteoarthritis: Rational approach to treating the individual. Best Pract. Res. Clin. Rheumatol..

[5] Smith G.K., Lawler D.F., Biery D.N., Powers M.Y., Shofer F., Gregor T.P., Karbe G.T., McDonald-Lynch M.B., Evans R.H., Kealy R.D. (2012). Chronology of hip dysplasia development in a cohort of 48 labrador retrievers followed for life. Vet. Surg..

[6] López M.J. (2012). Advances in hip dysplasia. Vet. Surg..

[7] Dassler C., Slatter D. (2003). Canine hip dysplasia: Diagnosis and nonsurgical treatment. Textbook of Small Animal Surgery.

[8] Henderson A., Millis D., Millis D., Levine D. (2004). Tissue Healing: Tendons, Ligaments, Bone, Muscles, and Cartilage. Canine Rehabilitation and Physical Therapy.

[9] Goldring M.B., Goldring S.R. (2007). Osteoarthritis. J. Cell. Physiol..

[10] Yun S., Ku S.K., Kwon Y.S. (2016). Adipose-derived mesenchymal stem cells and platelet-rich plasma synergistically ameliorate the surgical-induced osteoarthritis in Beagle dogs. J. Orthop. Surg. Res..

[11] Marx R.E. (2001). Platelet-rich plasma (PRP): What is PRP and what is not PRP?. Implant. Dent..

[12] Marx R.E., Carlson E.R., Eichstaedt R.M., Schimmele S.R., Strauss J.E., Georgeff K.R. (1998). Platelet-rich plasma: Growth factor enhancement for bone grafts. Oral. Surg. Oral. Med. Oral. Pathol. Oral. Radiol. Endod..

[13] Anitua E. (1999). Plasma rich in growth factors: Preliminary results of use in the preparation of future sites for implants. Int. J. Oral. Maxillofac. Implants.

[14] Canalis E. (1992). Clinical review 35: Growth factors and their potential clinical value. J. Clin. Endocrinol. Metab..

[15] Wu C.C., Chen W.H., Zao B., Lai P.L., Lin T.C., Lo H.Y., Shieh Y.H., Wu C.H., Deng W.P. (2011). Regenerative potentials of platelet-rich plasma enhanced by collagen in retrieving pro-inflammatory cytokine-inhibited chondrogenesis. Biomaterials.

[16] Civinini R., Nistri L., Martini C., Redl B., Ristori G., Innocenti M. (2013). Growth factors in the treatment of early osteoarthritis. Clin. Cases Miner. Bone Metab..

[17] Anitua E., Andia I., Sanchez M., Azofra J., del Mar Zalduendo M., de la Fuente M., Nurden P., Nurden A.T. (2005). Autologous preparations rich in growth factors promote proliferation and induce VEGF and HGF production by human tendon cells in culture. J. Orthop. Res..

[18] Okuda K., Kawase T., Momose M., Murata M., Saito Y., Suzuki H., Wolff L.F., Yoshie H. (2003). Platelet-rich plasma contains high levels of platelet-derived growth factor and transforming growth factor-beta and modulates the proliferation of periodontally related cells in vitro. J. Periodontol..

[19] Kawase T., Okuda K., Saito Y., Yoshie H. (2005). In vitro evidence that the biological effects of platelet-rich plasma on periodontal ligament cells is not mediated solely by constituent transforming-growth factor-beta or platelet-derived growth factor. J. Periodontol..

[20] Ha C.W., Park Y.B., Jang J.W., Kim M., Kim J.A., Park Y.G. (2019). Variability of the Composition of Growth Factors and Cytokines in Platelet-Rich Plasma from the Knee with Osteoarthritis. Arthroscopy.

[21] Forogh B., Mianehsaz E., Shoaee S., Ahadi T., Raissi G.R., Sajadi S. (2016). Effect of single injection of platelet-rich plasma in comparison with corticosteroid on knee osteoarthritis: A double-blind randomized clinical trial. J. Sports Med. Phys. Fitness.

[22] Vilar J.M., Manera M.E., Santana A., Spinella G., Rodriguez O., Rubio M., Carrillo J.M., Sopena J., Batista M. (2018). Effect of leukocyte-reduced platelet-rich plasma on osteoarthritis caused by cranial cruciate ligament rupture: A canine gait analysis model. PLoS ONE.

[23] Rodriguez-Merchan E.C. (2013). Intraarticular Injections of Platelet-rich Plasma (PRP) in the Management of Knee Osteoarthritis. Arch. Bone Jt. Surg..

[24] Hielm-Björkman A., Roine J., Lappalainen A., Junnila J., Laitinen-Vapaavuori O. (2012). An un-commissioned randomized, placebo-controlled double-blind study to test the effect of deep sea fish oil as a pain reliever for dogs suffering from canine OA. BMC Vet. Res..

[25] Eskelinen E.V., Liska W.D., Hyytiäinen H.K., Hielm-Björkman A. (2012). Canine total knee replacement performed due to osteoarthritissubsequent to distal femur fracture osteosynthesis: Two-year objective outcome. Vet. Comp. Orthop. Traumatol..

[26] Cuervo B., Rubio M., Sopena J., Dominguez J.M., Vilar J., Morales M., Cugat R., Carrillo J.M. (2014). Hip osteoarthritis in dogs: A randomized study using mesenchymal stem cells from adipose tissue and plasma rich in growth factors. Int. J. Mol. Sci..

[27] Shah K., Drury T., Roic I., Hansen P., Malin M., Boyd R., Sumer H., Ferguson R. (2018). Outcome of allogeneic adult stem cell therapy in dogs suffering from osteoarthritis and other joint defects. Stem Cells Int..

[28] Hunter D.J., Felson D.T. (2006). Osteoarthritis. BMJ.

[29] Wang-Saegusa A., Cugat R., Ares O., Seijas R., Cusco X., Garcia-Balletbo M. (2011). Infiltration of plasma rich in growth factors for osteoarthritis of the knee short-term effects on function and quality of life. Arch. Orthop. Trauma. Surg..

[30] Kazemi D., Fakhrjou A., Dizaji V.M., Alishahi M.K. (2014). Effect of autologous platelet rich fibrin on the healing of experimental articular cartilage defects of the knee in an animal model. Biomed. Res. Int..

[31] Millis D., Millis D., Levine D. (2004). Responses of Musculoskeletal Tissues to Disuse and Remobilization. Canine Rehabilitation and Physical Therapy.

[32] Slemenda C., Heilman D.K., Brandt K.D., Katz B.P., Mazzuca S.A., Braunstein E.M., Byrd D. (1992). Reduced quadriceps strength relative to body weight: A risk factor for knee osteoarthritis in women?. Arthritis Rheum..

[33] Hayes K.W., Falconer J. (1992). Differential muscle strength decline in osteoarthritis of the knee. A developing hypothesis. Arthritis Care Res..

[34] Monk M.L., Preston C.A., McGowan C.M. (2006). Effects of early intensive postoperative physiotherapy on limb function after tibial plateau leveling osteotomy in dogs with deficiency of the cranial cruciate ligament. Am. J. Vet. Res..

[35] Baltzer W.I., Smith-Ostrin S., Warnock J.J., Ruaux C.G. (2018). Evaluation of the clinical effects of diet and physical rehabilitation in dogs following tibial plateau leveling osteotomy. J. Am. Vet. Med. Assoc..

[36] Shires P.K., Braund K.G., Milton J.L., Liu W. (1982). Effect of localized trauma and temporary splinting on immature skeletal muscle and mobility of the femorotibial joint in the dog. Am. J. Vet. Res..

[37] Horstam C.L., Conzemius M.G., Evans R. (2004). Assessing the efficacy of perioperative oral carprofen after cranial cruciate surgery using noninvasive, objective pressure platform gait analysis. Vet. Surg..

[38] Tano C.A., Cockshutt J.R., Dobson H., Miller C.W., Holmberg D.L., Taves C.L. (1998). Force plate analysis of dogs with bilateral hip dysplasia treated with a unilateral triple pelvic osteotomy: A longterm review of cases. Vet. Comp. Orthopaed..

[39] Lopez S., Vilar J.M., Rubio M., Sopena J.J., Santana A., Rodriguez O., Rodriguez-Altonaga J.A., Carrillo J.M. (2019). Pedobarography: A novel approach to test the efficacy of treatments for lameness; an experience with mavacoxib in dogs with elbow osteoarthritis. BMC Vet. Res..

[40] Romano L.S., Cook J.L. (2015). Safety and functional outcomes associated with short-term rehabilitation therapy in the postoperative management of tibial plateau leveling osteotomy. Can. Vet. J..

[41] Alvarez L.X., Fox P.R., Van-Dyke J.B., Grigsby P. (2016). Survey of referring veterinarians perceptions of and reasons for referring patients to rehabilitation facilities. J. Am. Vet. Med. Assoc..

[42] Vilar J.M., Morales M., Santana A., Spinella G., Rubio M., Cuervo B., Cugat R., Carrillo J.M. (2013). Controlled, blinded force platform analysis of the effect of intraarticular injection of autologous adipose-derived mesenchymal stem cells associated to PRGF-Endoret in osteoarthritic dogs. BMC Vet. Res..

